# DNA Methylation and Transcriptome Profiling Reveal the Role of the Antioxidant Pathway and Lipid Metabolism in *Plectropomus leopardus* Skin Color Formation

**DOI:** 10.3390/antiox14010093

**Published:** 2025-01-15

**Authors:** Yang Liu, Linna Wang, Zhentong Li, Linlin Li, Tangtang Ding, Shuai Chen, Pengfei Duan, Xinyi Wang, Yishu Qiu, Xiaoyu Ding, Yongsheng Tian

**Affiliations:** 1 State Key Laboratory of Mariculture Biobreeding and Sustainable Goods, Yellow Sea Fisheries Research Institute, Chinese Academy of Fishery Sciences, Qingdao 266071, China; yangliu@ysfri.ac.cn (Y.L.); wangln@ysfri.ac.cn (L.W.); lizt@ysfri.ac.cn (Z.L.); lill@ysfri.ac.cn (L.L.); dingtangtang0928@163.com (T.D.); fisherman923@163.com (S.C.); duanpf_gs@163.com (P.D.); wxy229746642@163.com (X.W.); yishuqiu97@163.com (Y.Q.); dingxy0503@163.com (X.D.); 2 Key Laboratory for Sustainable Development of Marine Fisheries, Ministry of Agriculture and Rural Affairs, Yellow Sea Fisheries Research Institute, Qingdao 266071, China; 3 Hainan Innovation Research Institute, Chinese Academy of Fishery Sciences, Sanya 572025, China; 4 College of Fisheries, Tianjin Agricultural University, Tianjin 300392, China

**Keywords:** *Plectropomus leopardus*, body color, methylome, transcriptome, antioxidant pathway, lipid metabolism

## Abstract

Leopard coral grouper (*Plectropomus leopardus*), possessing a distinct red body color, is an important species in commercial markets; however, the high ratio of black individuals under intensive cultivation has limited the commercial value of the species. To dissect the regulatory mechanisms underlying the red skin trait in *P. leopardus*, gene expression and DNA methylation modifications were compared between red and black skin tissues after astaxanthin treatment. Astaxanthin effectively increased the redness value a* and body weight. Multi-omics analyses revealed the crucial roles of pathways related to antioxidants and lipid metabolism, particularly “Tyrosine metabolism”, “Melanogenesis”, “Fatty acid metabolism”, “Fatty acid elongation”, and “Biosynthesis of unsaturated acids”, in red skin coloration. A molecular network for the regulation of red skin coloration in *P. leopardus* was constructed, and *pmel*, *tyr*, *tyrp1a*, *tyrp1b*, *dct*, *slc24a5*, *wnt1*, *acsl4*, *elovl1*, *elovl6l.1*, *elovl6l.2*, and *elovl7* were identified as key genes. Notably, *pmel*, *acsl4*, and *elovl7* were negatively regulated by differential DNA methylation. Our results provide new insight into the molecular and epigenetic mechanisms of body color variation, representing a significant step towards breeding for the red skin trait in *P. leopardus*.

## 1. Introduction

Owing to its high commercial value, leopard coral grouper (*Plectropomus leopardus*) is widely cultured in many Asian countries. *P. leopardus* has a bright red skin color, a sign of auspiciousness in Chinese traditional culture; thus, the price of fish with red skin is 1.5–2 times that of individuals of other colors in the market [1,2]. However, the ratio of red-skinned individuals is quite low under intensive cultivation. Therefore, it is necessary to determine the mechanism underlying red skin coloration and improve the frequency of this trait in *P. leopardus* breeding.

The type and content of pigments, such as melanin, carotenoids, and pteridine, determine body color in fish and are related to genetics, dietary factors, and environmental conditions [3]. Previous studies have indicated that teleost fish possess six pigment cell types, including melanophores (black or brown), xanthophores (ocher or yellow), erythrophores (red), iridophores (iridescent), leucophores (white), and cyanophores (blue) [4]. The melanin level determines skin color in *P. leopardus* [5]. Melanin is synthesized in the melanophores/melanocytes through the specific lysosome-related organelles termed melanosomes [6]. Melanosomes are usually categorized into four stages (I–IV) based on the grade of morphological maturity; therein, melanin production and massive deposition occurs in the third and fourth stages [7]. In melanocytes, melanin production is mainly regulated by four categories of factors, involving enzymatic components for melanin synthesis (TYR, TYRP1, and TYRP2/DCT), fibrillar proteins involved in melanosome structure (PMEL, MART-1, and GPNMB), dynamic proteins for melanosome transport and transfer (APs, OA1, BLOC-1, and Rab27a), and melanogenic transcription factors (CREB, MITF, PAX3, SOX9, and SOX10) [4,7]. In addition, several key genes regulating melanin production and distribution have been identified through functional experiments. For example, mutations in *slc24a5* or *slc45a2* affect body color or lead to albinism in teleost fish [4,8]. However, the causative mechanisms of color variation under aquaculture conditions have not been clarified in most cultured fish.

Carotenoids serve as regulators of red and yellow phenotypes; they cannot be synthesized de novo in fish and are acquired through dietary sources [9]. Astaxanthin, a ketocarotenoid, is commonly used in aquaculture to promotes red body coloration and to improve antioxidant activity and immunity [3]. Furthermore, the light spectrum and tank color can directly affect body color and color-related gene expression in fish species [10,11,12]. There is a wealth of genomic resources on skin color variation in *P. leopardus*, including whole-genome sequencing [13,14,15,16], whole-transcriptome (mRNA, miRNA, circRNA, and lncRNA) [17,18,19,20,21,22,23], metabolomics [24], and proteomics data [25] as well as data from genome-wide association studies (GWASs) [1,26] and studies of functional genes [9,17]. However, red skin coloration in *P. leopardus* is determined by complex interactions among multiple regulatory mechanisms. The species can switch between red- or black-colored bodies under various aquaculture conditions. Here, we evaluated whether environmental factors, such as dietary astaxanthin, shape color-related gene transcription via DNA methylation. Briefly, DNA methylation is an important epigenetic modification and affects gene expression in response to environmental stimuli [27]. Depending on the visual environment, male cichlid fish (*Astatotilapia burtoni*) can change their body color from yellow to blue by downregulating the expression of the *ednrb* gene through DNA hyper-methylation within its promoter [28]. The role of DNA methylation in red body coloration has also been reported in common carp (*Cyprinus carpio*) and crucian carp (*Carassius carassius*) [29,30]. However, its contribution to coloration in *P. leopardus* has not been determined.

The primary objective of this study was to explore the epigenetic mechanisms underlying body color differences among *P. leopardus* individuals to aid the selection of high-value broodstock. We compared red and black skin tissues induced by astaxanthin treatment using RNA-seq and whole-genome DNA methylation analyses. The results highlight the vital role of DNA methylation in regulating red skin coloration. Furthermore, a potential regulatory network was constructed based on the color-related candidate genes. This study provides valuable data for uncovering the molecular and epigenetic mechanisms of red skin formation in *P. leopardus*.

## 2. Materials and Methods

### 2.1. Resources and RNA Extraction

**Figure 1:**
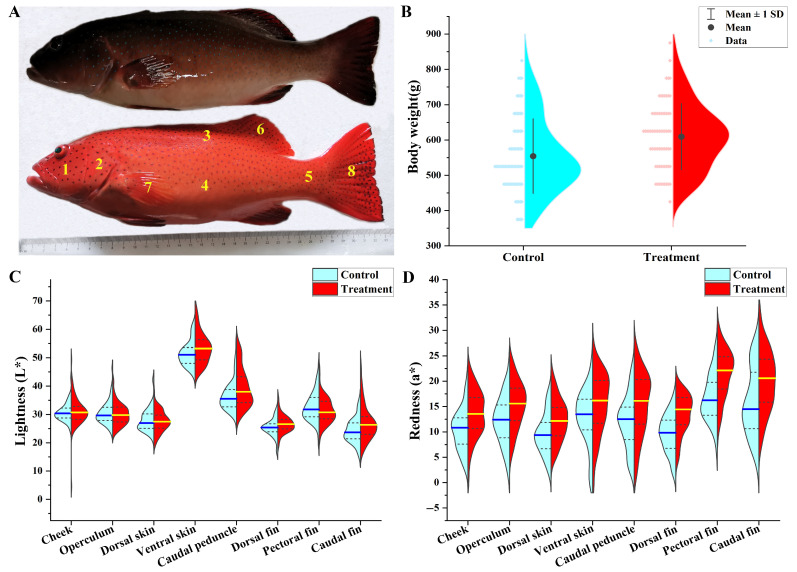
Comparison of growth and body color traits between groups. (**A**) Appearance and measured areas of *P. leopardus* in the control group (CB) and the treatment group (TR). The numbers 1–8 represent the cheek, operculum, dorsal skin, ventral skin, caudal peduncle, dorsal fin, pectoral fin, and caudal fin. (**B**) Comparison of body weight between the CB and TR groups. (**C**) Skin lightness in the CB and TR groups. (**D**) Skin redness in the CB and TR groups. The blue and yellow horizontal lines in subfigures C, D represent the mean values, whereas the dashed line indicates 1 standard deviation (SD).

The feeding experiment was carried out at Laizhou Ming Bo Aquatic Co., Ltd. (Laizhou, China) in water recirculation conditions. The larvae were derived from mixed broodstocks from a culture facility; these broodstocks have not been selected for body color traits. All larvae were raised on a compound pelleted diet (Santong Bioengineering Co., Ltd., Weifang, China) until reaching one year old. Then, healthy juveniles (356.76 ± 6.96 g) were randomly allocated into two groups, in triplicate, with each tank (10 m^3^) containing 300 fish. Commercial pellets supplemented with 0.10 g/kg astaxanthin were used for the treatment group (TR) and the control group (CB) was fed the diet without any carotenoid pigment. Natural astaxanthin for the feeding experiment was derived from *Haematococcus pluvialis* (3% purity), with the optical isomers of (3*S*, 3’*S*). After the astaxanthin was dissolved in water, it was evenly sprayed on the base feed and then dried naturally. The supplemental dosing and usage of astaxanthin were based on previous studies [31,32]. All culture tanks were identical and possessed a white bottom and walls. The light source was white LED lamps, and the light intensity and photoperiod were set to 1600 lux and 14:10 light:dark (L:D), respectively. The fish were fed twice a day (8 a.m. and 4 p.m.) at 2% of their body weight for 3 months, with a feed conversion ratio of 1.5. After the feeding trial, 70 individuals in two groups were measured for growth performance and colorimetric evaluation, respectively. The 3nh NR60CP Colorimeter (Guangdong Sanenshi Intelligent Technology Co., Ltd., Guangzhou, China) was used to measure the L* (the brightness) and a* (the redness) values on different body areas of the fish (Figure 1A). All measurements were performed following a previously described method [3]. Finally, the red and black dorsal skins were sampled from nine fish in two groups, and samples from three fish were combined to obtain a mixed sample for mRNA and DNA methylation analyses. Total RNA was extracted from the samples using the TRIzol reagent kit (Invitrogen, Waltham, MA, USA), adhering to the manufacturer’s protocol.

### 2.2. Transcriptome Analysis

The Agilent 2100 Bioanalyzer (Agilent Technologies, Santa Clara, CA, USA) and a 1% agarose gel were used for assessing RNA purity and integrity. RNA-seq libraries were prepared using the NEBNext Ultra RNA Library Prep Kit for Illumina and sequenced on the Illumina NovaSeq6000 platform (Gene Denovo Biotechnology Co. Ltd., Guangzhou, China). To obtain clean reads, adapters and low-quality sequences were filtered via fastp v0.18.0 with default parameters and then mapped to the reference genome [16] using Hisat2 v2.1.0. Fragments per kilobase of transcript per million mapped reads (FPKM) values were calculated using RSEM software package v1.3.3. Differentially expressed genes (DEGs) were screened using the DESeq2 R software 1.44.0 [33], with threshold values of FDR < 0.05 and |log_2_FC| ≥ 1. The DEG functions were further explored through Gene Ontology (GO) and Kyoto Encyclopedia of Genes and Genomes (KEGG) enrichment analyses.

### 2.3. Methylome Analysis

The samples for RNA-seq were resubmitted for the whole-genome bisulfite sequencing (WGBS). High-quality DNA was fragmented and purified using the MiniElute PCR Purification Kit (QIAGEN, Valencia, CA, USA). After end repair, poly(A) addition, adapter ligation, bisulfite conversion, and PCR amplification, the products were sequenced using the Illumina HiSeq 2500 (Gene Denovo Biotechnology Co., Guangzhou, China). After filtering, the clean reads were mapped to the reference genome [16] using BSMAP v2.90 [34]. A Perl script was used for calling the methylated cytosines [35]. For CG, CHG, and CHH contexts, genomic DNA methylation levels were evaluated according to the ratio of methylated cytosine on various gene regions. MethylKit v1.7.10 was used to identify differentially methylated regions (DMRs), with a sliding window of 200 bp according to the following criteria: (1) for CG and CHG, the numbers in each window ≥5, absolute difference in methylation ratio ≥0.20, and *q* ≤ 0.05; (2) for CHH, numbers in a window ≥10, absolute difference in methylation ratio ≥0.10, and *q* ≤ 0.05. Then, DMR-related genes (DMGs) were further analyzed through a KEGG pathway enrichment analysis.

### 2.4. Integrated Analysis of the Methylome and Transcriptome

Gene expression levels were categorized into four classes: non-expressed (RPKM ≤ 1), low expression (1 < RPKM ≤ 10), intermediate expression (10 < RPKM ≤ 100), and high expression (RPKM > 100). The region-wise method was used for quantifying DNA methylation levels on various genomic regions. Correlations between methylation and expression levels in gene regions (gene body ± 2 kb flanking regions) were calculated using Spearman’s correlation analyses. A positive correlation was confirmed by rho > 0, while rho < 0 represented a negative correlation.

Overlap between DMGs and DEGs was obtained, and overlapping genes were evaluated through a KEGG enrichment analysis.

### 2.5. Quantitative Real-Time Polymerase Chain Reaction (qRT-PCR)

To validate the accuracy of the transcriptome data, 14 DEGs involved in melanin synthesis and lipid metabolism were adopted for qRT-PCR verification. The cDNA was reverse-transcribed from total RNAs using the PrimeScript™ RT Reagent Kit (Takara Bio, Kusatsu, Japan). qRT-PCR was conducted in triplicate, and *β-actin* was used as a reference gene. qRT-PCR was performed using the LightCycler480II (Roche, Basel, Switzerland) with TB Green Premix Ex Taq II (Tli RNaseH Plus) (Takara Bio, Kusatsu, Japan). The reaction mixture was incubated for 30 s at 95 °C, followed by 40 amplification cycles of 5 s at 95 °C and 30 s at 60 °C. To ensure a single amplification reaction, a melting curve analysis was conducted at the end of each run. The 2^−ΔΔCt^ method was used for calculating the relative gene expression levels. The primers used for validation are listed in Table S1.

### 2.6. Data Analysis

Statistical analyses were carried out using one-way analysis of variance (ANOVA) with Duncan’s multiple comparison tests, via IBM SPSS Statistics 26.0 (SPSS Inc., Chicago, IL, USA). The experimental data are expressed as the mean ± standard deviation (SD), and *p* < 0.05 was considered statistically significant.

## 3. Results

### 3.1. Growth and Colorimetric Evaluation

After 3 months of the feeding trial, body weight was significantly higher in the TR group (609.33 ± 11.25 g) than in the CB group (554.26 ± 12.67 g) (Figure 1B). Similarly, the condition factor was significantly higher in the TR group (3.46 ± 0.04%) than in the CB group (3.30 ± 0.03%).

The color parameters L* and a* in different body areas are shown in Figure 1C,D. The brightness (L*) value was higher on the ventral skin than on other body areas. Furthermore, the L* value was significantly higher in the TR group than in the CB group on the ventral skin, caudal peduncle, dorsal fin, and caudal fin. The redness (a*) value was highest on the pectoral fin. Notably, the values of a* in TR were higher than those in CB in all eight test areas (Figure 1D). In particular, the fin rays, including the dorsal, pectoral, and caudal fins, showed substantially higher redness values in TR.

### 3.2. Identification of DEGs and Functional Enrichment Analysis

**Figure 2:**
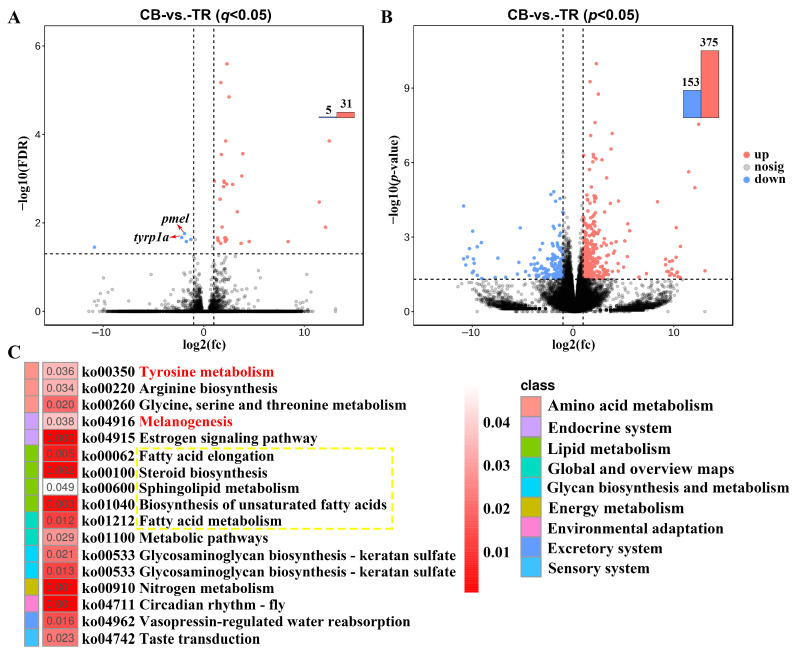
Differentially expressed genes (DEGs) and KEGG enrichment analysis. (**A**) DEGs in skin tissue between groups with the threshold of *q* < 0.05. (**B**) DEGs in skin tissue between groups with the threshold of *p* < 0.05. (**C**) KEGG analysis of the DEGs between groups. Red indicates pathways involved in melanin synthesis. The yellow box represents DEGs involved in lipid-associated functional pathways.

After filtering, a total of 45.41 Gb of clean data and 301.30 M clean reads were obtained. Detailed information on sequence data is presented in Supplementary Table S2. The ratios of total reads mapped ranged from 88.77% to 91.38%, with an average value of 90.67%. A total of 36 DEGs were identified between TR and CB with the threshold of *q* < 0.05, including 31 upregulated and 5 downregulated genes in TR. Notably, the melanin synthesis genes *tyrp1a* and *pmel* were significantly downregulated in the astaxanthin-treated group (Figure 2A).

To illustrate the potential regulatory network, 528 DEGs were further identified with a threshold of *p* < 0.05, of which 375 and 153 were up- and downregulated, respectively (Figure 2B). A KEGG enrichment analysis revealed that the candidate DEGs were significantly involved in “Tyrosine metabolism” and “Melanogenesis”. Furthermore, enrichment for lipid-metabolism-associated function, including “Fatty acid elongation”, “Biosynthesis of unsaturated acids”, “Sphingolipid metabolism”, and “Fatty acid metabolism”, was detected (Figure 2C).

**Figure 3:**
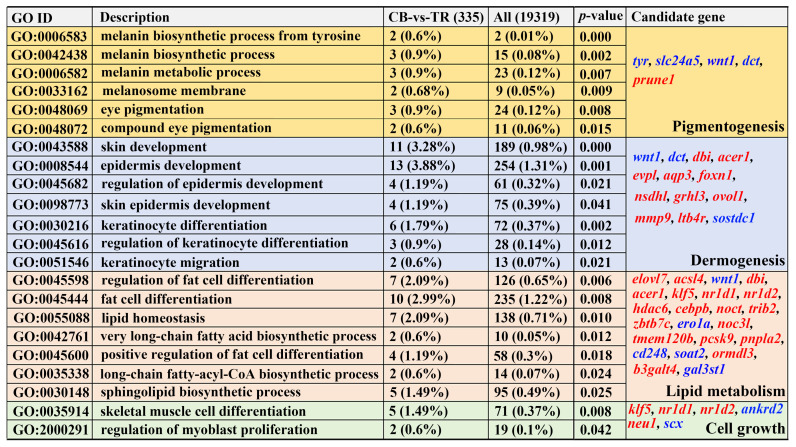
GO enrichment analysis of DEGs related to body color. Red and blue represent up- and downregulated genes, respectively. The different background colors represent pathways involved in pigmentogenesis, dermogenesis, lipid metabolism, and cell growth.

A GO enrichment analysis revealed significant enrichment for terms related to pigmentogenesis and dermogenesis, such as “Melanin biosynthetic process”, “Melanin metabolic process”, “Eye pigmentation”, “Epidermis development”, and “Keratinocyte differentiation”. The melanin synthesis genes *tyr*, *slc24a5*, *wnt1*, and *dct* were all downregulated (Figure 3). Furthermore, there was enrichment for terms related to lipid metabolism and cell growth, including “Fat cell differentiation”, “Lipid homeostasis”, and “Long-chain fatty-acyl-CoA biosynthetic process”, consistent with the KEGG pathway enrichment results. Moreover, numerous lipid metabolism genes exhibited elevated expression levels in the red skin group, such as *elovl7*, *acsl4*, *dbi*, *acer1*, *klf5*, *nr1d1*, and *nr1d2* (Figure 3).

### 3.3. Identification of DMGs and Functional Enrichment Analysis

The resulting clean reads totaled 0.78 Gb, with a sequencing depth of 26.77 times and 74.05% genome mapping rate (Table S3). In both groups, the mCpG type was identified as the major type, accounting for 95% of mCs, whereas mCHG and mCHH accounted for 3.14% and 0.89%, respectively (Figure S1A). Individuals of the same color showed higher similarity in DNA methylation patterns, as identified through a Pearson correlation analysis (Figure S1B). Genomic DNA methylation levels did not show significant differences between the TR and CB groups. Furthermore, the genomic DNA methylation levels were lower in the regions 2 kb upstream of genes across both test groups (less than 50%) (Figure S1C). DNA methylation levels were further analyzed in different genomic regions. For mCHG and mCHH contexts, higher methylation levels were broadly detected in different gene regions in the TR group than in the CB group. However, for CpG sites, there was a slight decrease in methylation levels in gene body, intron, and downstream 2 kb regions and a mild increase in the other gene regions (Figure S1C).

The CpG sites possessed the most DMRs (8734), whereas there were fewer DMRs (24 and 156) at CHG and CHH sites, respectively. Further genomic annotation of DMRs revealed 3360 DMGs, of which 1563 and 1797 showed hyper- and hypo-methylation. A KEGG enrichment analysis revealed numerous pathways associated with pigmentogenesis, such as “Melanoma”, “EGFR tyrosine kinase inhibitor resistance”, and “Wnt signaling pathway” (Figure S2).

### 3.4. Correlations Between DNA Methylation and Gene Transcript Levels

**Figure 4:**
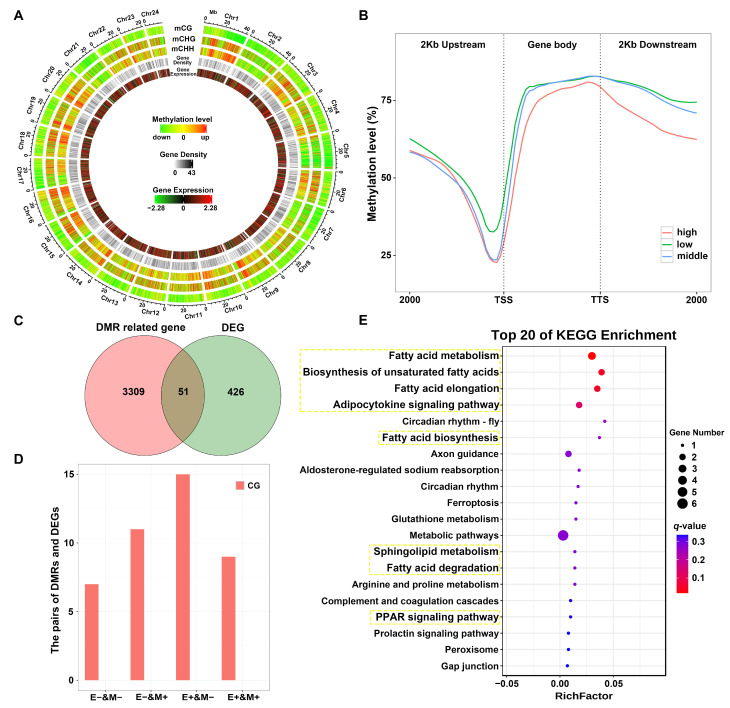
Correlation analysis between DNA methylation and gene transcript levels and KEGG pathway enrichment. (**A**) Differential methylation and expression distributions at the chromosomal level. (**B**) Trends in gene expression and DNA methylation within the same genomic regions. (**C**) Venn diagram of DMGs and DEGs. (**D**) Correlations between DMGs and DEGs. (**E**) KEGG pathway enrichment analysis of the overlapping genes. The yellow box represents the common genes enriched in lipid-associated pathways.

Compared with the control group, genomic regions with differential methylation and expression levels were detected at the chromosomal level in the TR group (Figure 4A). A negative correlation between gene transcript levels and DNA methylation was detected in all gene regions across both test groups (Figure 4B). In total, 51 common genes between DEGs and DMGs were extracted (Figure 4C). Twenty-four common genes exhibited a negative correlation between gene expression and DNA methylation. Among these, 11 genes were downregulated with DNA hyper-methylation, while 13 genes were upregulated, exhibiting hypo-methylation. Furthermore, “E+ & M−” and “E− & M+” were identified as the main types of DNA methylation responsible for DEGs (Figure 4D).

A KEGG analysis of the common genes showed enrichment for lipid-metabolism-related pathways (Figure 4E), including “Fatty acid metabolism”, “Biosynthesis of unsaturated fatty acids”, “Fatty acid elongation”, “Adipocytokine signaling pathway”, “Fatty acid biosynthesis”, “Sphingolipid metabolism”, “Fatty acid degradation”, and “PPAR signaling pathway”. The lipid synthesis genes *acsl4*, *elovl6l.1*, and *elovl7* were upregulated. Notably, *acsl4* and *elovl7* exhibited DNA hypo-methylation in the CB vs. TR comparison.

### 3.5. Lipid Metabolism Played a Vital Role in Red Skin Coloration

**Figure 5:**
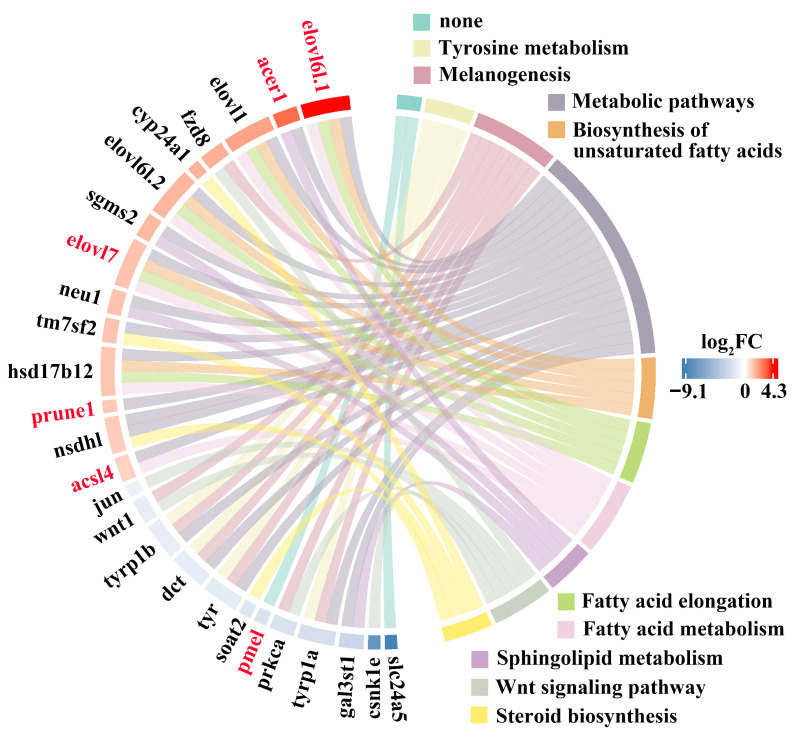
Melanin-synthesis- and lipid-metabolism-associated DEGs identified in the skin tissue. Red represents DNA methylation regulation. Boxes of different colors represent melanin-synthesis- and lipid-related pathways.

The transcriptomic and methylomic analyses together highlighted the vital role of lipid-metabolism-related categories, such as “Biosynthesis of unsaturated fatty acids”, “Fatty acid elongation”, “Fatty acid metabolism”, and “Sphingolipid metabolism”, in red skin formation. Color ribbons were used to demonstrate the inclusion relation between the candidate genes and functional pathways. Interestingly, numerous candidate genes were upregulated and were modulated by DNA methylation, including *elovl6l.1*, *elovl7*, *acsl4*, and *acer1* (Figure 5). In contrast, melanin synthesis pathways were extensively downregulated, such as “Tyrosine metabolism”, “Melanogenesis”, and “Wnt signaling pathway”. These genes involved in black skin coloration were significantly downregulated, including *pmel*, *tyr*, *tyrp1a*, *tyrp1b*, *dct*, *slc24a5*, and *wnt1*. Notably, the *pmel* gene, required proper melanosome development, was downregulated by DNA hyper-methylation (Figure 5).

**Figure 6:**
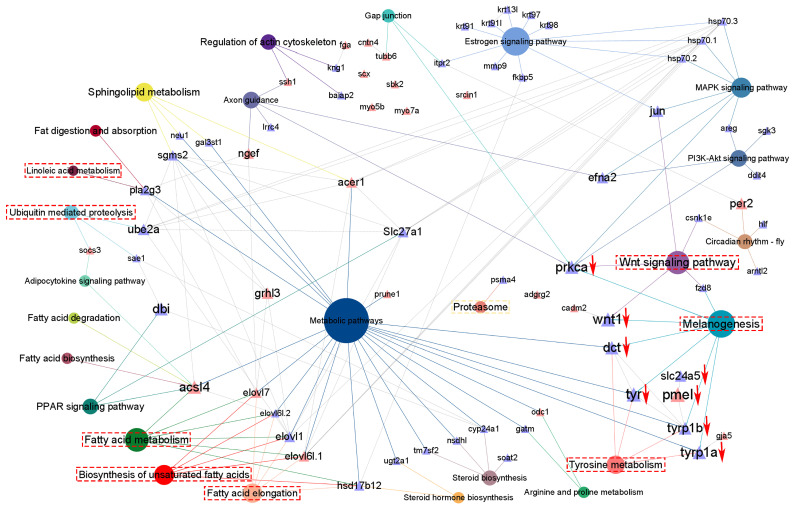
Interaction analysis between candidate pathways and genes involved in body color formation. Triangles and circles indicated candidate genes and functional pathways. The red triangle represents DNA methylation regulation. The red arrow represents downregulated melanin-synthesis-associated genes. The dashed red boxes highlight the key pathways. The connection lines correspond to the color of the pathway, indicating that the gene was enriched in this pathway.

According to candidate gene expression patterns, an interaction analysis was carried out to construct a potential molecular regulatory network for pigmentogenesis. In the network, *acsl4*, *prkca*, *ube2a*, *acer1*, and *slc27a1* were identified as hub genes (Figure 6). Interestingly, melanin synthesis genes were broadly downregulated, and their potential regulatory relationships based on expression data were identified among *tyr*, *dct*, *tyrp1b*, *pmel*, and *slc24a5* (Figure 6).

**Figure 7:**
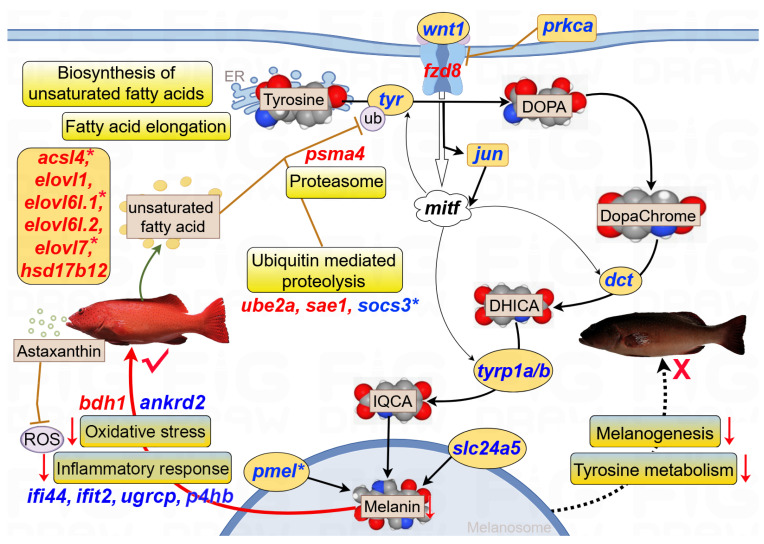
Potential regulatory network based on candidate genes for body color traits of *P. leopardus*. Key pathways included “Oxidative stress”, “Inflammatory response”, “Unsaturated fatty acid synthesis”, “Ubiquitin mediated proteolysis”, “Proteasome”, “Tyrosine metabolism”, and “Melanogenesis”. Red and blue gene names indicated up- and downregulation, respectively. The star * represented DNA methylation.

Based on the above bioinformatics analysis and manual literature searches, a potential molecular network for the role of lipid metabolism in the regulation of red skin coloration was constructed for *P. leopardus* (Figure 7). This regulatory network contained “Astaxanthin intake”, “Unsaturated fatty acid synthesis”, “Tyrosinase degradation”, and “Melanin synthesis”. Notably, inflammatory- and stress-associated genes (*ifi44*, *ifit2*, *ugrcp*, *ankrd2*, *p4hb*, and *bdh1*) were mostly downregulated in the TR group. However, genes involved in “Biosynthesis of unsaturated fatty acids” and “Fatty acid elongation” were upregulated, which might facilitate the synthesis of unsaturated fatty acids. Consequently, unsaturated fatty acids decreased melanin synthesis through promoting tyrosinase degradation; this process was mediated by proteasome and ubiquitination, and related regulatory genes were mostly upregulated, including *psma4*, *ube2a*, and *sae1*. “Melanin synthesis” pathways, including “Tyrosine metabolism” and “Melanogenesis”, were downregulated in the red skin group. Furthermore, the key genes *pmel*, *acsl4*, *elovl6l.1*, *elovl7*, and *socs3* were also regulated by DNA methylation. The regulatory mechanisms of candidate genes and pathways in body color traits are highlighted in the Discussion section.

### 3.6. Validation of DEGs by qRT-PCR

**Figure 8:**
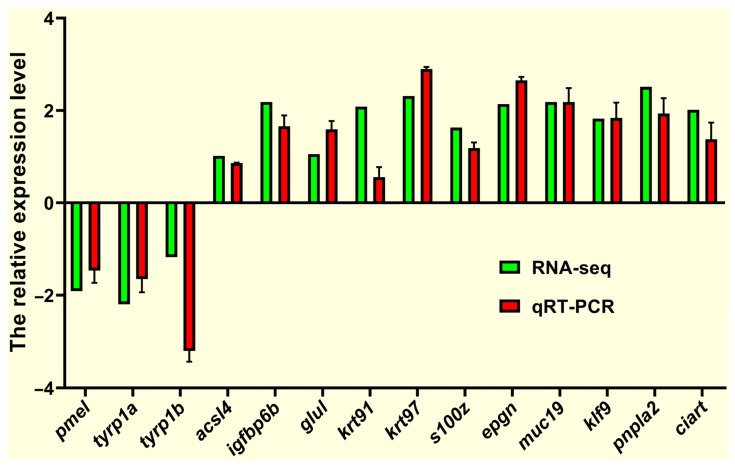
Verification of RNA-seq data using qRT-PCR. *β-actin* was set as the reference gene to calculate the expression levels of target genes.

To evaluate the accuracy of RNA-seq, we selected 14 DEGs (*pmel*, *tyrp1a*, *tyrp1b*, *acsl4*, *igfbp6b*, *glul*, *krt91*, *krt97*, *s100z*, *epgn*, *muc19*, *klf9*, *pnpla2*, and *ciart*) for validation using qRT-PCR, as summarized in Figure 8. The *pmel*, *tyrp1a*, and *tyrp1b* genes, involved in melanin synthesis, were downregulated, consistent with the RNA-seq results. The *acsl4* gene, involved in lipid metabolism, was significantly upregulated in both analyses. In short, the overall strong concordance between qRT-PCR and RNA-seq results underscored the reliability of the findings.

## 4. Discussion

The red body color trait is increasingly utilized in *P. leopardus* breeding. However, a low frequency of red individuals has been found in artificial cultivation, possibly due to environmental factors, such as astaxanthin supplementation. Therefore, understanding the molecular and epigenetic mechanisms underlying red body color formation is essential for supporting *P. leopardus* breeding. In this study, a combined transcriptome and methylomics analysis was used to clarify the mechanism underlying skin pigmentation caused by astaxanthin in *P. leopardus* cultivation.

### 4.1. Astaxanthin Increases Body Weight and Redness

After 3 months of cultivation, the body weight was higher in the TR group than in the CB group. This suggested that astaxanthin addition exerted a beneficial effect on growth, consistent with previous results for *P. leopardus* [3]. The addition of astaxanthin to the diet is widely used in aquaculture, and the roles of astaxanthin in fish growth, immunity, and antioxidant activity have been verified by phenotypic assays, histological observations, enzyme activity assays, transcriptome analyses, and studies of functional genes [3,36]. Multiple studies have demonstrated that astaxanthin can reduce lipid accumulation and enhance fish growth through promoting the absorption and utilization of metabolic intermediates in nutrients [36,37].

Moreover, astaxanthin addition effectively increased the redness value (a*) compared with that in the CB group across all test areas in *P. leopardus*. Under environmental stimulus, the a* value decreased more significantly in body areas than in the fin rays. Therefore, high red differences were detected in each tested fin ray, with the highest value observed for the pectoral fin. Accordingly, these findings suggest that the pectoral fin should be used as a representative test area for the redness a* of *P. leopardus*. However, there was little difference in the brightness L* in numerous test areas, contrary to previous findings showing significant reductions in the L* value in the TR group [3]. This difference among studies may be related to differences in the developmental stage and testing environment.

### 4.2. Transcriptome and Methylome Patterns in P. leopardus

In a transcriptome analysis, a total of 36 DEGs were identified between the TR and CB groups. Notably, the melanin synthesis genes *tyrp1a* and *pmel* were markedly downregulated in the TR group, similar to previous results [3,24]. The identified DEGs were significantly enriched in amino acid metabolism, endocrine system, and lipid metabolism categories and were involved in vital pathways, such as “Tyrosine metabolism”, “Melanogenesis”, “Fatty acid elongation”, “Biosynthesis of unsaturated acids”, and “Fatty acid metabolism”.

Although DNA methylation, as a key epigenetic regulation, participates in various biological processes, few studies have focused on its role in fish pigmentation through the regulation of target genes. Thus, a WGBS analysis was used to evaluate the red and black skin of *P. leopardus*. A total of 8734 DMRs were found, including 1563 hyper- and 1797 hypo-methylated DMGs. These DMGs were involved in pathways related to pigmentogenesis, such as “Melanoma”, “EGFR tyrosine kinase inhibitor resistance”, and “Wnt signaling pathway”. The pivotal role of “Wnt signaling pathway” in coloration has also been demonstrated in DNA methylation analysis of honey bees [38].

Highly expressed genes showed reduced DNA methylation in different gene regions. This negative correlation was weak in the 2 kb upstream regions, which may be explained by lower levels of genomic methylation in these regions [39]. In gene bodies, DNA hypomethylation plays a vital role in regulating transcript elongation and alternative splicing [40]. Moreover, “E+ & M−” and “E− & M+” were identified as the main types of DNA methylation responsible for DEGs. These results revealed that DNA methylation contributes to body color variation in *P. leopardus* through regulating inducible and reversible gene expression.

The common genes between DEGs and DMGs were enriched for lipid metabolism pathways, such as “Fatty acid metabolism”, “Biosynthesis of unsaturated fatty acids”, “Fatty acid elongation”, “Fatty acid biosynthesis”, and “PPAR signaling pathway”. Notably, the lipid synthesis genes *acsl4*, *elovl6l.1*, and *elovl7* were upregulated, and *acsl4* and *elovl7* exhibited DNA hypo-methylation. Previous transcriptome analyses of *P. leopardus* body color have yielded similar results, that is, candidate DEGs were specifically enriched in lipid-related pathways [3,24,36]. Notably, *acsl4* could regulate melanin synthesis through activating ferroptosis (promoting cell death via lipid peroxidation) [2].

### 4.3. Melanin Synthesis Inhibition Might Promote Red Coloration

Two types of chromatophores, light-reflecting iridophores and aggregated light-absorbing melanophores, were identified in the skin of *P. leopardus*, in the upper and lower layers of the dermis, respectively [41]. The body color of *P. leopardus* might change from red to black or vice versa depending on the melanosome count and distribution. Black body colors occur because melanosomes extend their dendritic processes to cover iridophores and absorb light. Only the light reflected by iridophores is visible, and the body color turns red [41]. Melanin is synthesized in the melanophores determining the skin color of *P. leopardus* [5]. Solar ultraviolet radiation and inflammatory stimuli promote melanin biosynthesis via increasing the level of intracellular reactive oxygen species (ROS) [42]. Therefore, we hypothesized that intensive cultivation caused oxidative stress and inflammatory responses in *P. leopardus*, ultimately leading to an increase in the proportion of black individuals [43]. Similarly, the melanin content was over eight-fold higher in the dorsal skin of farmed red porgy (*Pagrus pagrus*) than in wild individuals [44].

Antioxidants, such as astaxanthin, protect fish cells against oxidative damage [45]. Previous transcriptome studies have reported that astaxanthin supplementation reduces fat accumulation and oxidative damage in the liver of *P. leopardus* [36]. Intriguingly, genes involved in inflammatory- and stress-responsive functions were mostly downregulated in the TR group. Interferon-induced protein 44 (IFI44) is a potential prognostic indicator in the inflammatory response [46]. During oxidative stress caused by the exposure of myoblasts to H_2_O_2_, *Ankrd2* expression is slightly upregulated via ROS [47]. Through targeting P4HB, salidroside, a main active ingredient of *Rhodiola*, could inhibit inflammation and melanogenesis [48]. BDH1 over-expression could inhibit the excessive production of ROS, protecting HK-2 cells from glucotoxicity and lipotoxicity via the NRF2-mediated antioxidant pathway [49]. The *bdh1* gene was significantly upregulated in the TR group. Therefore, we speculated that astaxanthin treatment attenuated the inflammatory response and oxidative stress, thereby inhibiting melanin biosynthesis via decreasing levels of ROS [50].

### 4.4. Signaling Lipids Play a Vital Role in Body Color Formation

For melanin synthesis, tyrosinase (TYR), as a rate-limiting enzyme, catalyzes tyrosine to generate 3,4-dihydroxyphenylalanine (DOPA) and further oxidizes DOPA to dopaquinone. Tyrosinase-related protein 2 (TYRP2/DCT) catalyzes dopachrome to DHI-2-carboxylic acid (DHICA). Under the regulation of TYRP-1, DHICA produces indole-5,6-quinone-2-carboxylic acid (IQCA), thereby generating eumelanin (Figure 7). Therefore, it is generally believed that the concentration of melanin is directly proportional to TYR expression and activity [51,52]. Intriguingly, the catalytic genes (*tyr*, *dct*, *tyrp1a*, and *tyrp1b*) were consistently downregulated in the TR group.

Unsaturated fatty acids, such as linoleic acid and docosahexaenoic acid (DHA), can inhibit melanin synthesis through increasing TYR degradation, which is regulated by the ubiquitin–proteasome system [52,53]. Long-chain polyunsaturated fatty acids are key components of lipid molecules and are biosynthesized through fatty acyl desaturases (Fads) and elongation of very-long-chain fatty acid (Elovl) proteins. The candidate *elovl* genes (*elovl1*, *elovl6l.1*, *elovl6l.2*, and *elovl7*) were upregulated in the TR group. Moreover, *elovl6l.1* and *elovl7* were regulated by DNA methylation. DHA, as the longest and the least saturated fatty acid, can decrease melanin production via the promotion of TYR degradation in murine melanoma cells [52]. In vertebrates, astaxanthin can improve the stability of DHA by inhibiting oxidation of non-esterified polyunsaturated fatty acids [54]. The condition factor was higher in the TR group than in the CB group. Therefore, we speculated that astaxanthin promoted the synthesis of unsaturated fatty acids, thereby inhibiting melanin biosynthesis through increasing TYR degradation. Furthermore, the *tyrp2* gene was identified in a GWAS analysis of the black color trait in *P. leopardus* and possessed the most prominent marker, SNP1733237 [26]. Thus, the findings provided crucial targets for marker-assisted selection and gene editing for body color traits in *P. leopardus*.

## 5. Conclusions

The study used a combined transcriptomics and methylomics approach to elucidate the genetic and molecular basis of body color formation in *P. leopardus*. Astaxanthin addition effectively increased the redness a* and body weight in the TR group. Furthermore, the antioxidant pathway and lipid metabolism were strongly related to red skin coloration. Finally, candidate genes were highly enriched for “Tyrosine metabolism”, “Melanogenesis”, “Fatty acid metabolism”, “Fatty acid elongation”, and “Biosynthesis of unsaturated acids”. These results contribute to the dissection of mechanisms underlying body color formation, thus facilitating molecular breeding for the red skin trait in *P. leopardus*.

## Data Availability

The transcriptome and methylome data presented in the study are deposited in the NCBI SRA repository, accession numbers PRJNA1189492 and PRJNA1189689, respectively.

## Supplementary Materials

The following supporting information can be downloaded at: https://www.mdpi.com/article/10.3390/antiox14010093/s1, Figure S1: The genomic DNA methylation patterns in the black and red skin tissues of *P. leopardus*; Figure S2: KEGG enrichment analysis of differentially methylated genes; Table S1: The primers used in the present study; Table S2: The reads information in the whole-transcriptome analysis; Table S3: The sequencing data information in the WGBS.
