# Supplementary material for: DNA Methylation and Transcriptome Profiling Reveal the Role of the Antioxidant Pathway and Lipid Metabolism in Plectropomus leopardus Skin Color Formation
Source: Antioxidants (Basel). 2025 Jan 15;14(1):93. doi: 10.3390/antiox14010093 (PMC11763275; doi:10.3390/antiox14010093)
Supplement: Supplementary file 1 [file antioxidants-14-00093-s001.zip › Supplementary Figure.docx]

**Figure S1.** The genomic DNA methylation patterns in the black and red skin tissues of *P. leopardus*.

(A) The proportion of different types of DNA methylation, including mCG, mCHG, and mCHH. (B) The clustering analysis of CpG methylation in different samples. (C) The total DNA methylation levels of mCG, mCHG, and mCHH in different genomic regions. CB and TR represented the control group and the treatment group, respectively.


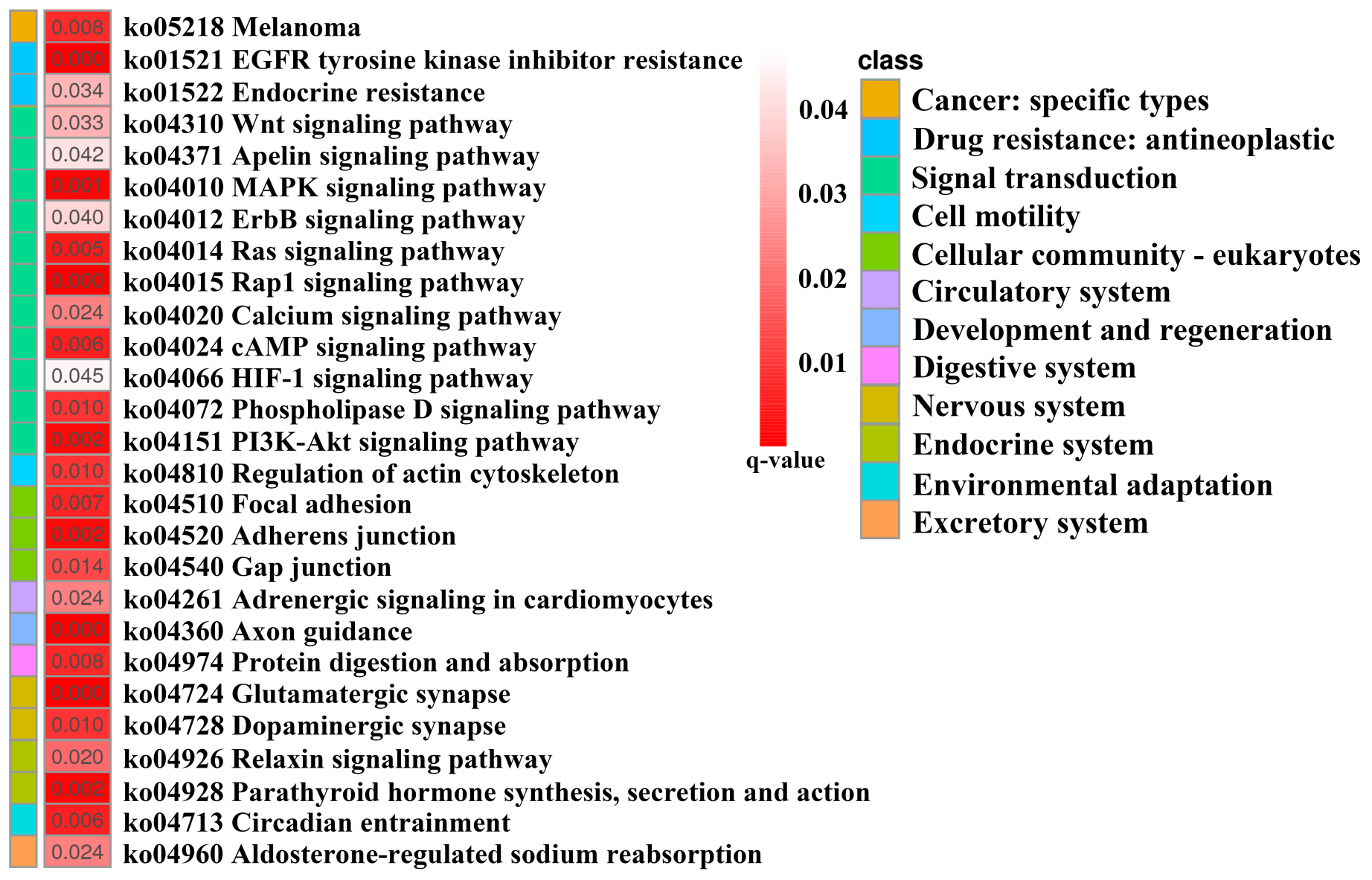


**Figure S2.** KEGG enrichment analysis of differentially methylated genes.
